# Exploring Mental Health Status and Syndrome Patterns Among Young Refugee Children in Germany

**DOI:** 10.3389/fpsyt.2018.00212

**Published:** 2018-05-25

**Authors:** Thimo Buchmüller, Hanna Lembcke, Julian Busch, Robert Kumsta, Birgit Leyendecker

**Affiliations:** ^1^Department of Developmental Psychology, Faculty of Psychology, Ruhr-University Bochum Bochum, Germany; ^2^Department of Genetic Psychology, Faculty of Psychology, Ruhr-University Bochum Bochum, Germany

**Keywords:** refugees, specificity, mental health, early childhood, prevalence, PTSD, CTRF, CBCL

## Abstract

Refugee children share a large number of pre-, peri-, and post-migration risk factors, which make them vulnerable for developing mental health concerns. Within the last few years, a large number of families with young children have sought refuge in Germany. However, children's mental health status in Germany is mostly unclear. A central aim of developmental psychopathology is to understand how risk factors lead to the emergence of mental health concerns. One approach to investigating this association is the study of specificity, which describes the idea that specific risk factors are related to specific psychological outcomes. The aim of our study was to assess the mental health status of young refugee children in Germany, and to explore a potential refugee-specific mental health pattern. In two studies, we assessed mental health outcomes of 93 children from Syria or Iraq, aged 1.5–5 years, who recently arrived in Germany. The results were compared to U.S. norm data of typically developing children, and to norm data of a clinical sample in order to explore mental health patterns. In the first study (*n* = 35), we used standardized screening tools for parents (CBCL 1.5-5). In the second study (*n* = 58), mental health states of refugee children were assessed by caretakers (CTRF 1.5–5). In comparison to U.S. norm data of normally developing children, refugee parents reported more mental health concerns for their children, especially on syndrome scales of internalizing difficulties. A comparison to U.S. clinical reference data showed a specific mental health pattern, characterized by increased levels of anxiety/depression, attention problems, and withdrawal behavior. Caretakers, too, reported more mental health problems compared to typically developing children, albeit to a smaller extent. However, a comparison to clinically-referred children only led to partial confirmation of a specific mental health pattern. Our studies offer important insights into the mental health status and pattern of young refugee children, which is essential for preventing the onset of psychopathology and for offering tailored interventions.

## Introduction

In 2015 alone, 65.3 million people, half of them being children, were forcibly displaced worldwide. This represents the highest number of forced displacement documented in history (1). The majority of people sought refuge in the context of the Syrian war (2). Germany became a prominent recipient of new asylum applications, with about 745,000 requests in 2016 (3). Thus, the significance of physical and mental health needs of refugee families gained great attention within the German health care system.

A review by Fazel et al. (4) organizes the negative factors that affect children's mental health outcomes chronologically by stage in the migration process. Risk factors during pre-migration include organized violence and the loss of family members. Peri-migration risk factors encompass disruption of schooling, lack of security, and material poverty. Post-migration risk factors include stigmatization, language barriers, acculturation difficulties, frequent relocations, low socio-economic status, as well as insecurity regarding residence permissions. Even if refugees and immigrants share some risk-factors, the number, severity, and kind of common risks within the refugee population go beyond those immigrants are exposed to. Thus, refugees present a “unique population” in need of tailored research and interventions (5).

Considering the number of risk factors, a negative impact of forced displacement on psychological outcomes can be expected. Indeed, studies on refugees' mental health status report elevated prevalence rates of depression, anxiety, and post-traumatic stress disorder (PTSD) in refugee children as compared to non-refugee children (6). A small, but growing number of studies investigate the mental health status of children in the context of the Syrian war. Children, ages 4–10, were screened in refugee camps near the Turkish-Syrian border (7). According to parent-reports, these children showed increased levels of fear (62%), anxiety and withdrawal (49%), emotional problems (45%), conduct problems (38%), and hyperactivity (20%). Another study conducted in a refugee campsite at the Syrian-Turkish border reported that the vast majority of children had experienced more than one critical life event, such as the loss of a family member (8). In fact, 44% of the children reported five or more war-related critical life events. Furthermore, 45% showed signs of PTSD. Similarly, 44% of the children showed signs of mood disorders, 20% had manifested clinical depression, and 25% reported somatic symptoms (e.g., stomach pain) on a daily basis. Overall, refugee children seem to have higher prevalence rates of mental health concerns. These findings are also supported by studies that investigated mental health outcomes among young refugees in Germany (9–11).

While some studies reported an effect on a wide array of mental health domains (6, 8, 9), other studies found a specific effect concerning symptoms of PTSD (10, 11). Specificity describes the idea that specific risk factors are uniquely related to specific psychological outcomes (12). Evidence remains inconsistent about the influence of experiencing war-related and migration-related violence and deprivation in early childhood on specific outcomes. A large scale retrospective survey showed that emotional neglect in childhood was specifically associated with affective disorders in adulthood (13). After controlling for comorbidities and adversities Becker-Blease et al. (14) found that young children exposed to victimization (e.g., abuse) manifested more mental health concerns than those experiencing disasters (e.g., flood) in a representative U.S. sample. A review by McMahon et al. (12) subsumes 16 studies which examined the influence of violence on psychological outcomes. Different types of violence were assessed, including domestic violence, war, and community violence. Outcomes included PTSD, depression, general anxiety, aggression symptoms, and internalizing and externalizing symptoms. Half of the studies did not report any evidence for specificity, whereas eight found evidence for specificity. Five of the eight studies reported a specific association between violence exposure and externalizing outcomes, whereas three studies reported a link between the exposure to violence and internalizing behavior. However, the review did not distinguish between types of violence nor did it include war-related violence.

A study by Hodges et al. (15) showed an association between the number of traumatic events and complexity of symptomatology. That is, within a clinical sample of children between 8 and 12 years of age, those children who were exposed to multiple traumatic events demonstrated a higher complexity of symptoms (15). However, only few studies investigated mental health patterns of persons who were exposed to war-related violence. One study showed that comorbidity between PTSD and mood disorder was 13.5 times higher for adult Palestinians who had experienced violence than for Palestinians who had not experienced violence (16). Refugee school children, between 6 and 16 years of age, who were interviewed shortly after the 1994 siege of Sarajevo, showed differences in mental health adjustments depending on the type of violence they were exposed to. Children who were exposed to deprivation and violence showed more withdrawn behavior and anxiety problems compared to children who experienced violence only (17). Hence, a certain type of violence may lead to a specific pattern of syndromes, which might be characterized by a higher symptom complexity.

The present study fills the gap of missing data on mental health outcomes of recently arrived refugee children below 6 years of age. Research on refugee children in this age group is scarce. Considering that these children were exposed to risk factors specific to their population (5), it is likely that they manifest specific mental health patterns. The aim of our study was to assess a variety of behavioral symptoms that are typical of young refugee children who have experienced traumatization and deprivation. Applying an explorative approach, we focused on two research questions: What is the mental health status of young refugee children in Germany compared to typically developing children? Do symptom manifestations differ from clinically-referred children, which might indicate a refugee-specific mental health pattern?

We applied a three-step approach in order to investigate our research questions. First, we conducted a pilot study to assess the feasibility of screening instruments, and to get first insights into the prevalence rates of mental health concerns of this specific population of young, recently arrived refugee children. The findings of the pilot study were used to design Study 1. In Study 1, we interviewed refugee parents from Syria and Iraq rather than relying on questionnaires. In Study 2, we included a large sample of caretakers from early childhood education and care centers for recently arrived refugee children. As caretakers might have a different perspective on children's behavior, Study 2 was used as a triangulation. The results obtained were compared to norm data of typically developing children and to norm data of children with clinical manifestations. We report the results of the pilot study, and Studies 1 and 2 separately, before integrating the results into the overall discussion.

## Pilot study

### Methods

#### Participants

A convenience sample was used. We collected data from 48 children. Due to missing values, and age beyond the range covered by the instruments, 17 children were excluded. The final sample size consisted of 31 children (Age: *M* = 2.9; *SD* = 1.3; female: 56%). Parents had, on average, 10.3 years of education (*SD*: 5.0 years). While most children came from Syria (92%), there were a few from Iraq (8%).

#### Materials

The Child Behavior Checklist [CBCL 1.5–5 Arabic translation in Lebanese dialect, which is similar to Syrian dialect, a Syrian version was not available; (18)] was administered to measure children's mental health status as reported by mothers. The instrument is validated for children from 1.5 to 5 years of age. The 100 items can be grouped into eight syndrome scales. Four of them (Emotionally Reactive, Anxious/Depressed, Somatic Complaints, Withdrawn) can be combined into the broadband scale of Internalizing Behavioral Problems; two (Aggressive Behavior, Attention Problems) can be combined into the broadband scale of Externalizing Behavioral Problems. Additionally, the CBCL has a scale for Sleep Problems and a scale for Other Problems. Furthermore, a selection of specific items of the CBCL can be used to estimate if a child suffers from PTSD (19). It is considered to be a cost-effective and valid screening tool for the presence of PTSD symptoms in preschool children.

The respondents indicate on a 3-point Likert scale if a problem is either not true (0), somewhat or sometimes true (1), or very true or often true (2), based on the child's behavior over the previous 2 months. Norms of typically developing children and matched clinically-referred children who were considered for mental health services are available from 18. Norms are based on a representative sample from U.S. children and differentiate between boys and girls. While scores are controlled for gender, age shows no effect on the syndrome scales according to the manual. As U.S. children and Arabic children from the United Arab Emirates generally show similar manifestations on CBCL scales, the use of U.S. norms can be considered to be cross-culturally valid (20).

#### Procedure

In the context of an evaluation project, a team of research assistants visited 44 primary childcare projects (*Bridging Projects*, German: *Brückenprojekte*) designated for refugee families recently arrived in the German federal state of North-Rhine Westphalia in 2016 and 2017. Projects were free of charge and either in refugee centers or in close proximity in order to provide easy access for families. As enrollment opportunities in regular primary care centers are scarce, this low-threshold approach was implemented by the government to serve as temporary compensation. Caretakers were asked to distribute informed consent forms and questionnaires to Arabic speaking refugee families from either Syria or Iraq, whose children were aged 1.5-5. Subsequently, families passed questionnaires back to the caretakers who returned them to us. The response rate was 29%, i.e., we received less than a third of our distributed questionnaires by the end of the study. Written informed consent was obtained by every respondent who filled out the questionnaire. The study was approved by the Ethics Committee of the Faculty of Psychology of the Ruhr-University Bochum.

#### Statistical analysis

Data were processed in SPSS® 25.0 for Windows (IBM Corporation, Armonk, NY, USA). We excluded all cases that had missing values in more than 10% of the CBCL items. For all other cases, missing values were replaced by the mean of the item across participants. Clinical and subclinical cutoff scores were calculated according to the manual (18). The clinical cutoff for the broadband scales was set at two standard deviations above the average score of the norm data of typically developing children. We used the PTSD scale and the proposed cutoff of 9 points to estimate the frequency of children showing signs of PTSD (19).

Effect sizes (Cohen's *d*, 95% Confidence interval) were calculated comparing the syndrome manifestations of refugee children to typically developing and clinical-referred children (18), respectively. Effect size calculations were conducted using a syntax by Wuensch (21). When effect sizes of refugee children compared to clinical children were similar (using the 95% confidence interval), or refugee children had higher values, we additionally looked at comorbidities between syndrome scales by applying subclinical cutoffs (18). Effect size calculations enable score standardization. Hence, scores between syndrome scales were made comparable.

#### Results

On average, refugee children showed elevated prevalence rates on the total score (48%), the internalizing scale (48%), and the externalizing scale (48%). They showed elevated prevalence rates in three out of seven syndromes (Anxious/Depressed: 10%, Withdrawn: 20%, Attention Problems: 23%), whereas prevalence rates in the four remaining scales were in the normal range (Somatic Complaints: 3%; Aggressive Behavior: 7%; Sleep Problems: 0%, Emotionally Reactive: 3%). Similar rates can be found applying sub-clinical cutoffs: 19% of the children showed Anxiety/Depression in a subclinical range, 23% Somatic Complaints, 32% Withdrawal, 10% Attention Problems, 10% Aggressive Behavior, 7% Sleep Problems, and 13% Emotional Reactivity. One fifth of the children were above the cutoff on the PTSD scale (PTSD: 20%).

Refugee children had higher manifestations on syndrome scales compared to typically developing children, except for Emotional Reactivity, which only showed a minor mean difference (Anxious/Depressed: *M* = 1.8, *SD* = 3.0, *d* = 0.6 [CI 95%: 0.2; 1.0], Somatic Complaints: *M* = 1.1, *SD* = 1.9, *d* = 0.6 [CI 95%: 0.2; 0.9], Withdrawn: *M* = 1.7, *SD* = 2.9, *d* = 0.6 [CI 95%: 0.2; 1.0], Aggressive Behavior: *M* = 0.5, *SD* = 2.9, *d* = 0.1 [CI 95%: −0.3; 0.4], Attention Problems: *M* = 2.2, *SD* = 3.0, *d* = 0.7 [CI 95%: 0.3; 1.1], Emotionally Reactive: *M* = 0.0, *SD* = 2.9, *d* = 0.0 [CI 95%: −0.3; 0.4]).

Scores on Anxiety/Depression and Attention Problems were also more pronounced compared to norm data of clinically-referred children, whereas Withdrawal was within the clinical range (Anxious/Depressed: *M* = 0.6, *SD* = 3.0, *d* = 0.2 [CI 95%: −0.2; 0.5], Attention Problems: *M* = 0.8, *SD* = 3.0, *d* = −0.3 [CI 95%: −0.6; 0.1], Withdrawn: *M* = −0.7, *SD* = 2.9, *d* = −0.2 [CI 95%: −0.6; 0.1]. All other syndrome manifestations were substantially lower in the refugee group than in the clinical group (Emotional Reactive: *M* = −2.3, *SD* = 2.9, *d* = −0.8 [CI 95%: −1.2; −0.4], Somatic Complaints: *M* = −2.0, *SD* = 3.0, *d* = −1.1 [CI 95%: −1.5; −0.6], Sleep Problems: *M* = −1.6, *SD* = 2.1, *d* = −0.7 [CI 95%: −1.1; −0.3], Aggressive Behavior: *M* = −4.2, *SD* = 7.8, *d* = −0.5 [CI 95%: −0.9; −0.2]).

The comorbidity patterns of elevated syndrome scales in the refugee group (Anxious/Depressed, Withdrawn, Attention Problems) revealed that 16% of the refugee children fell above subclinical cutoffs on all three syndrome scales, and 10% fell above the cutoff on two syndromes (3% Anxious/Depressed & Attention Problems, 7% Withdrawn & Attention Problems). In contrast, 17% of the children fell above the cutoff on only one scale (10% on Withdrawn, 7% on Attention Problems, 0% on Anxious/Depressed).

#### Discussion

In line with a previous study on adolescent refugees in Germany (9), we found a high number of refugee children showing a total symptom score in a clinical range. Another recent study that assessed PTSD by using a structured interview with parents of very young children also showed increased rates of clinical symptoms (11). We gained a first insight into the pattern of mental health concerns consisting of anxiety/depression, withdrawal, and attention problems. Interestingly, the comorbidity of all three syndromes was high (16%). Do the three syndromes constitute a clinical syndrome pattern that might be characteristic of vulnerable refugee children? This question cannot be answered at the present stage, but should be addressed in future studies.

Unfortunately, demographic information regarding gender of the child, or the family's current living situation was missing in many cases. The feasibility of a questionnaire study in this population might be restricted. Anecdotal reports from educators and Arabic interpreters suggest that parents often have low literacy skills. Moreover, parents often refused participation because of a lack of trust in institutions. This might have led to a selection bias, as parents with a higher education might have been more likely to participate.

Although results have to be interpreted with care as the sample size was limited and background information was missing, they offer a first hint into the prevalence rates of mental health issues among young refugee children. Moreover, the comorbidity pattern and the elevated syndrome manifestations on three scales point to a potential refugee-specific mental health pattern. However, an interview format might ensure a higher degree of feasibility. This avoids a potential selection bias based on literacy and education.

## Study 1

### Methods

#### Participants

The inclusion criteria were: (1) fluent in Arabic language, (2) country of origin is Syria or Iraq (3) flight within past 3.5 years, (4) child between 1.5 and 5 years of age. Parents were the respondents in all cases (Mother: *n* = 27, Father: *n* = 3).

We collected data on 30 children. Demographic information about the children and the family is shown in Table 1. The families of the children came from Syria (*n* = 27), and Iraq (*n* = 3). All responding parents shared one household with their partners and children. On average, families had three children. Data were only obtained for one child per family.

**Table 1:** Demographic information of children and families in Study 1 (*n* = 35).

**Demographic variables**	***M* (*SD*)**
Current age of child (years)	3.7 (1.3)
Gender of child: Girls	18 (51)
Age of parent	33.7 (7.0)
Number of children per family	2.9 (1.5)
Education of parent in years^a^	11.6 (4.6)
Age of child during flight (years)^b^	2.2 (1.3)
Duration of flight (months)^c^	11.9 (14.7)
Time in Germany (months)^d^	18.4 (9.7)
Number of mothers pregnant during flight *n* (%)^e^	9 (26)

*M, mean; SD, standard deviation; n, number of cases*.

a *Number of years spent at school, including higher education (n = 29)*.

b *Age of the child when mother left home town in cases where the child was already born (n = 19)*.

c *Time span from leaving home town to arriving in Germany (n = 31)*.

d *Time span from arriving in Germany to interview (n = 31)*.

e *Number of mothers pregnant with target child during flight (n = 31)*.

#### Materials

The CBCL 1.5-5 (Arabic translation) was used (see Pilot Study).

#### Procedure

To avoid the feasibility problems we observed in the pilot study, we decided to administer the CBCL in the form of an interview. This allowed comprehensibility in a population unfamiliar with psychological assessments and enabled us to introduce inclusion criteria to restrict our assessment to refugee families. The interview was conducted by Arabic-speaking research assistants, who were trained by a clinical psychologist and regularly supervised. All families were recruited through word of mouth by our Arabic speaking research assistants at mosques, cultural centers, refugee camps, and language schools. Interviews were conducted at participants' homes in order to ensure comfort and safety. In addition, interviewers spent an extended time period on introductions and small talk before starting the interview. Subsequently, our research assistants explained the procedure and the purpose of the interview. Afterwards, written informed consent was obtained from all respondents. Interviews included additional questionnaires and tests that are not part of this study. Research assistants estimated the CBCL part of the interview to last approximately 30 min. At the end of the interview, participants had the opportunity to ask questions, and research assistants handed out gift certificates as a gesture of appreciation for taking part in the study. The study was approved by the Ethics Committee of the Faculty of Psychology of the Ruhr-University Bochum.

#### Statistical analysis

We applied stricter exclusion criteria regarding missing data than in the Pilot Study. If more than 10% of the items were missing, cases were not considered for statistical analysis. Clinical and subclinical cutoffs were calculated, as in the Pilot Study. The normality assumption of the syndrome scales was explored by conducting a Kolmogorov-Smirnov test (*p*_*exact*_ < 0.05).

First, the syndromes scores were normalized by calculating difference scores between the refugee sample and the U.S. norm sample of typically developing children. This yielded an expected value of zero in case of equality of both groups. In a next step, a dependent one-sample *t*-test was performed. The difference value between refugees and the norm data of typically developing children was compared against zero (*p* < 0.05). We applied Bonferroni corrections in order to control for multiple comparison biases.

Second, we compared the refugee data to clinical norm data, repeating the aforementioned statistical steps. We expected the difference from the clinical norm to be low. Given our sample size, a 5% threshold would lead to low power [required sample size = 128, if *d* = 0.3, *c* = 5%, (22)]. To avoid an inflation of beta errors, we used effect sizes (Cohens' *d*) as decision criteria for specificity. If refugees had a) poorer mental health than the clinical group defined as a positive effect size or b) had equal mental health defined as the 95% confidence interval of the effect size ranging from a negative value to a positive value, we considered the syndrome to be refugee-specific.

Third, clustering of syndromes was explored using a Venn diagram. It depicts the percentage of children above the subclinical cutoff, and the comorbidity of the syndromes that were identified as being potentially refugee-specific.

#### Results

**Figure 1:**
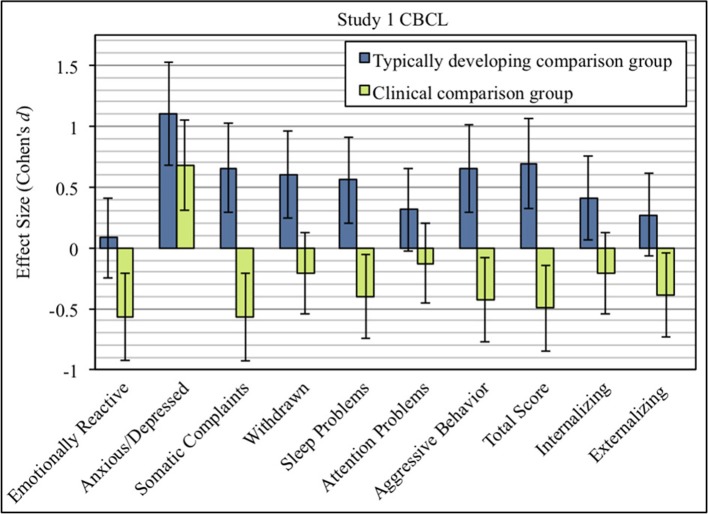
Effect sizes (Cohen's *d*) of CBCL syndrome scales comparing refugee children from Study 1 with typically developing children (blue), and clinically-referred children (green). Error bars indicate 95% confidence intervals of effect sizes.

On average, refugee children showed elevated total problem scores when compared to the U.S. norm of typically developing children. Means and inferential statistics are displayed in Table 2. Refugees scored higher on the internalizing scale, but did not differ significantly on the externalizing scale. As shown in Figure 1, they had elevated scores in four out of seven syndrome scales (Anxious/Depressed, Attention Problems, Withdrawn, Somatic Complaints).

**Table 2:** Study 1 CBCL syndromes of refugee children compared to norm data of typically developing children.

**CBCL syndromes**	***M* (*SD*) difference**	***t*-Values**	***p*-Values**	**Effect size *d* [CI 95%]**
Emotionally Reactive	0.3 (3.5)	0.5	1.0	0.1 [-0.3; 0.4]
Anxious/Depressed	3.1 (2.8)	6.5	< 0.01	1.1 [0.7; 1.5]
Somatic Complaints	1.7 (2.5)	3.9	< 0.01	0.7 [0.3; 1.0]
Withdrawn	1.8 (2.9)	3.6	0.01	0.6 [0.2; 1.0]
Sleep Problems	0.8 (2.9)	1.6	1.0	0.3 [-0.1; 0.6]
Attention Problems	1.1 (2.0)	3.3	0.02	0.6 [0.2; 0.9]
Aggressive Behavior	2.0 (6.4)	1.9	0.73	0.3 [0.0; 0.7]
Total Score	14.5 (22.2)	3.9	< 0.01	0.7 [0.3;1.0]
Internalizing	6.8 (9.9)	4.1	< 0.01	0.7 [0.3; 1.1]
Externalizing	3.1 (1.3)	2.4	0.21	0.4 [0.1; 0.8]

*CBCL, Child Behavior Checklist; M, mean; SD, standard deviation; n, number of cases; r, rank biserial correlations coefficient; d, Cohen's d; T-values for t-tests between refugee children and norm data of typically developing children (18), comparison of mean difference to 0; p-Values were adjusted for multiple comparisons using the Bonferroni-correction*.

Means and group comparisons with clinically-referred children, as well as prevalence rates of the refugee sample, are shown in Table 3. In general, refugee children showed lower psychological difficulties compared to norm data of clinically-referred children (see Figure 1). However, levels of behavioral difficulties varied between syndrome scales. Although refugee children had better mental health outcomes than the clinical norm group on most syndrome scales, differences were only marginal on the scales Attention Problems and Withdrawn. However, the effect size for the scale Anxious/Depressed was large, indicating that refugee children exhibited more behavior problems than clinically-referred children.

**Table 3:** Study 1 CBCL syndromes of refugee children compared to clinical norm data of clinically-referred children.

**CBCL syndromes**	***M* (*SD*) difference**	***t*-Values**	***p*-Values**	**Effect size *d* [CI 95%]**	**Clinical cutoff *n* (%)**	**Subclinical cutoff *n* (%)**
Emotionally Reactive	−2.0 (3.5)	−3.4	0.02	−0.6 [−0.2; −0.9]	3 (8.6)^a^	4 (11.4)^d^
Anxious/Depressed	1.9 (2.8)	4.0	< 0.01	0.7 [0.3; 1.0]	6 (17.1)^a^	15 (42.9)^d^
Somatic Complaints	−1.4 (2.5)	−3.4	0.02	−0.6 [−0.2; −0.9]	2 (5.7)^a^	9 (25.7)^d^
Withdrawn	−0.6 (2.9)	−1.3	1.0	−0.2 [0.1; −0.6]	8 (22.9)^a^	11 (31.4)^d^
sleep Problems	−1.2 (2.9)	−2.4	0.24	−0.3 [−0.1; −0.7]	3 (8.6)^a^	5 (14.3)^d^
Attention Problems	−0.3 (2.0)	−0.7	1.0	−0.1 [0.2; −0.5]	1 (2.9)^a^	10 (28.6)^d^
aggressive Behaviour	−2.7 (6.4)	−2.5	0.17	−0.4 [−0.1; −0.8]	0 (0)^a^	4 (11.4)^d^
Total Score	−11.0 (22.2)	−2.9	0.06	−0.5 [−0.1; −0.9]	22 (62.9)^b^	32 (91.4)^e^
Internalizing	−2.1 (9.9)	−1.2	1.0	−0.2 [0.1; −0.5]	9 (25.7)^b^	26 (74.3)^e^
Externalizing	−3.0 (7.6)	2.4	0.28	−0.4 [0.0; −0.7]	20 (57.1)^b^	28 (80.0)^e^
PTSD	–	–	–	–	13 (37)^c^	–

*CBCL, Child Behavior Checklist; PTSD, Post traumatic stress disorder; M, mean; SD, standard deviation; n, number of cases; d, Cohen's d; T-values for t-tests between refugee sample and clinical norm data (18), comparison of mean difference to 0; P-values were adjusted for multiple comparisons using the Bonferroni-correction*.

a *Number of refugee children who scored above clinical cutoffs as proposed by Achenbach and Rescorla (18)*.

b *Number of refugee children who scored at least two standard deviations above the norm (18)*.

c *Number of refugee children who scored above the cutoff proposed by Dehon and Scheeringa (19)*.

d *Subclinical cutoffs (18)*.

e *Number of refugee children who scored at least one standard deviation above the norm (18)*.

**Figure 2:**
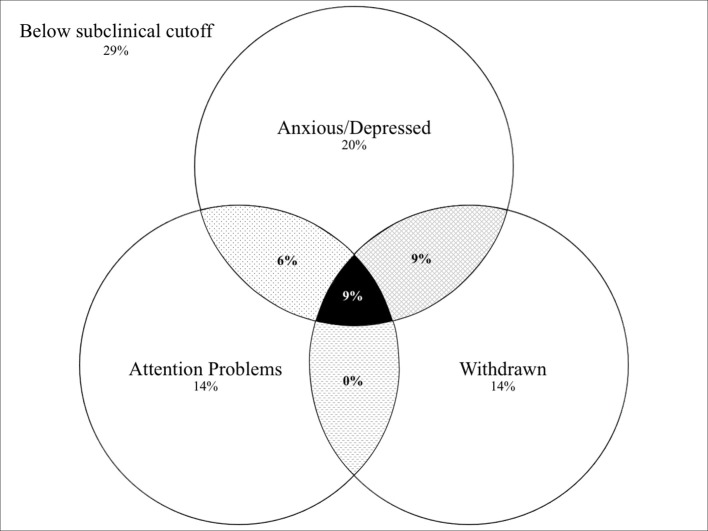
Percentage of refugee children of Study 1 that were above subclinical cutoff according to Achenbach and Rescorla (18) on identified potential refugee-specific syndromes (Attention problems, Anxious/Depressed, Withdrawn) and their comorbidities.

The comorbidity patterns of elevated syndrome scales in the refugee group (Anxious/Depressed, Withdrawn, Attention Problems) revealed that 9% of the refugee children fell above subclinical cutoffs on all three syndrome scales, while 15% were above the cutoff on two syndrome scales (see Figure 2). In contrast, the rest of children were above the cutoff on only one syndrome scale (10% on Withdrawn, 7% on Attention Problems, 0% on Anxious/Depressed).

#### Discussion

Compared to norm data of typically developing children, refugee children scored significantly higher on the scales Somatic Complaints, Attention Problems, Anxious/Depressed, and Withdrawn, which is indicated by large effect sizes. Somatic Complaints were less pronounced in refugee children than in clinically-referred children, but symptoms of Withdrawn, Attention Problems, and Anxious/Depressed were equal to or higher than in a clinical norm sample. Interestingly, the prevalence rates and affected syndromes in Study 1 bear a great resemblance to the findings of our pilot study. This consistency yields further evidence of the observed mental health status and potential pattern.

Data provide some indications for a refugee-specific mental health pattern. Data from Study 1 suggest that the combination of Withdrawn, Attention Problems, and Anxious/Depressed could potentially be regarded as a refugee-specific mental health pattern. As reported by parents, refugee children showed equal or higher levels of mental health problems on these three scales when compared to clinical norm data. Considering the clustering of these syndromes, they mostly occurred without a comorbid syndrome. However, 24% showed at least one comorbid syndrome within this potential refugee-specific pattern.

The elevated scores on specific syndrome scales on the CBCL are in line with mental health problems reported in previous studies on refugee children, e.g., altered anxiety levels, withdrawal, emotional problems, and attention problems (7). Most studies cluster the symptoms around a broad syndrome of traumatization and PTSD (8). In the present study, 37% of the children were above the proposed cutoff on the PTSD scale (19), making it likely that some of these children may suffer from PTSD. When compared to a clinical group, the different syndrome manifestations on some scales, in addition to the comorbidity of these syndromes found in a subgroup of refugee children, raise the question whether the observed mental health outcome indicates a refugee-specific mental health pattern rather than PTSD.

## Study 2

### Methods

#### Participants

The total sample of the second study comprised 59 children. One case was deleted because of missing data. Hence, the final sample consisted of 58 children. Of those, 47 families came from Syria and 11 from Iraq. There was no overlap between the children of Study 1 and Study 2. See Table 4 for demographic information.

**Table 4:** Demographic information of children (*n* = 58) in Study 2.

	***M* (*SD*)/*n* (%)**
Age in years	3.6 (1.2)
Girls	33 (57)
Time in Germany (months)^a^	19 (5.7)
Both parents present^b^	50 (86)
Literate parents^c^	49 (83)

*M, mean; SD, standard deviation; n, number of cases*.

a *Time span from arrival in Germany to participation in the study as estimated by the caretaker (n = 27)*.

b *Father and mother live in the household of the child estimated by the caretaker (n = 57)*.

c *Estimated by caretakers (n = 51)*.

#### Materials

The Caregiver Teacher Report Form [CTRF 1.5-5, German version; (18)] was administered to measure children's mental health status as reported by caretakers. The CTRF consists of 100 items, which largely correspond to the items on the CBCL. Caretakers also indicated their answers to 100 items on a 3-point Likert scale. Both instruments show a similar factor structure, have good content validity, and show a high cross-information agreement between parents and caretakers (18).

The CTRF has been a valid and reliable tool across countries (20). As for the CBCL, data of norm groups are available. The clinical reference group consists of children who were considered for mental health services and who were demographically matched to the typically developing norm group according to gender, ethnicity, age, and socio-economic background (18).

#### Procedure

Caretakers were recruited from early childcare centers for recently arrived refugee and immigrant children in the federal state of North Rhine-Westphalia in Germany. Parents were informed by caretakers about the purpose of the study prior to the assessments. Information was handed out in the form of a letter which was available in relevant languages, i.e., Arabic, English, and German. Parents who did not want their children to be screened for mental health outcomes were asked to inform caretakers within 1 week in order not to be considered for the assessment. Afterwards, caretakers were asked to complete the CTRF for the child, aged 1.5–5 years, they had known for the longest period of time. On average, caretakers indicated that they had known the child for 9.7 months (*SD* = 5.1). Personal information of the children and information of the care-centers were anonymized. The study was approved by the Ethics Committee of the Faculty of Psychology of the Ruhr-University Bochum.

#### Statistical analysis

We repeated the same statistical procedure as in the previous study. However, only two scales (Attention Problems and Aggressive Behavior) were approximately normally distributed, whereas the remaining scales deviated significantly from normality using the Kolmogorov-Smirnov test (*p*_*exact*_ < 0.05). Hence, we used non-parametric one-sample Wilcoxon tests for group comparisons.

#### Results

Refugee children had higher levels of externalizing problems than the norm. Attention Problems and Aggressive Behavior were elevated compared to U.S. norm data of typically developing children, although not in a significant range. Similarly, there were no differences in any other domains. Medians and group comparisons are shown in Table 5.

**Table 5:** Study 2 CTRF syndromes of refugee children compared to norm data of typically developing children.

**CTRF syndromes**	**Median (IQR) difference**	***z*-values**	***p*-values**	**Effect size *d* [CI 95%]**
Emotionally Reactive	−0.3 (3.3)	1.4	1.0	0.3 [0.1; 0.5]
Anxious/Depressed	−0.2 (4.2)	0.1	1.0	0.2 [−0.1; 0.5]
Somatic Complaints	−0.5 (1.2)	−0.3	1.0	0.1 [−0.0; 0.5]
Withdrawn	−1.3 (5.1)	0.2	1.0	0.2 [0.0; 0.5]
Attention Problems	0.9 (6.3)	2.6	0.10	0.4 [0.2; 0.7]
Aggressive Behavior	2.1 (13.0)	2.3	0.19	0.4 [0.2; 0.7]
Total Score	−0.1 (42.3)	1.8	0.71	0.3 [0.1; 0.6]
Internalizing	−1.4 (11.0)	0.5	1.0	0.2 [0.0; 0.5]
Externalizing	2.8 (17.2)	2.5	0.12	0.4 [0.2; 0.7]

*CTRF, Caretaker Teacher Report Form; M, mean; SD, standard deviation; n, number of cases; d, Cohen's d; Standardized z-values for one-sample Wilcoxon-test between refugee children and norm data of typically developing children (18), comparison of median difference to 0; P-Values were adjusted for multiple comparisons using the Bonferroni correction; Confidence interval was calculated using the syntax of Wuensch (21)*.

**Figure 3:**
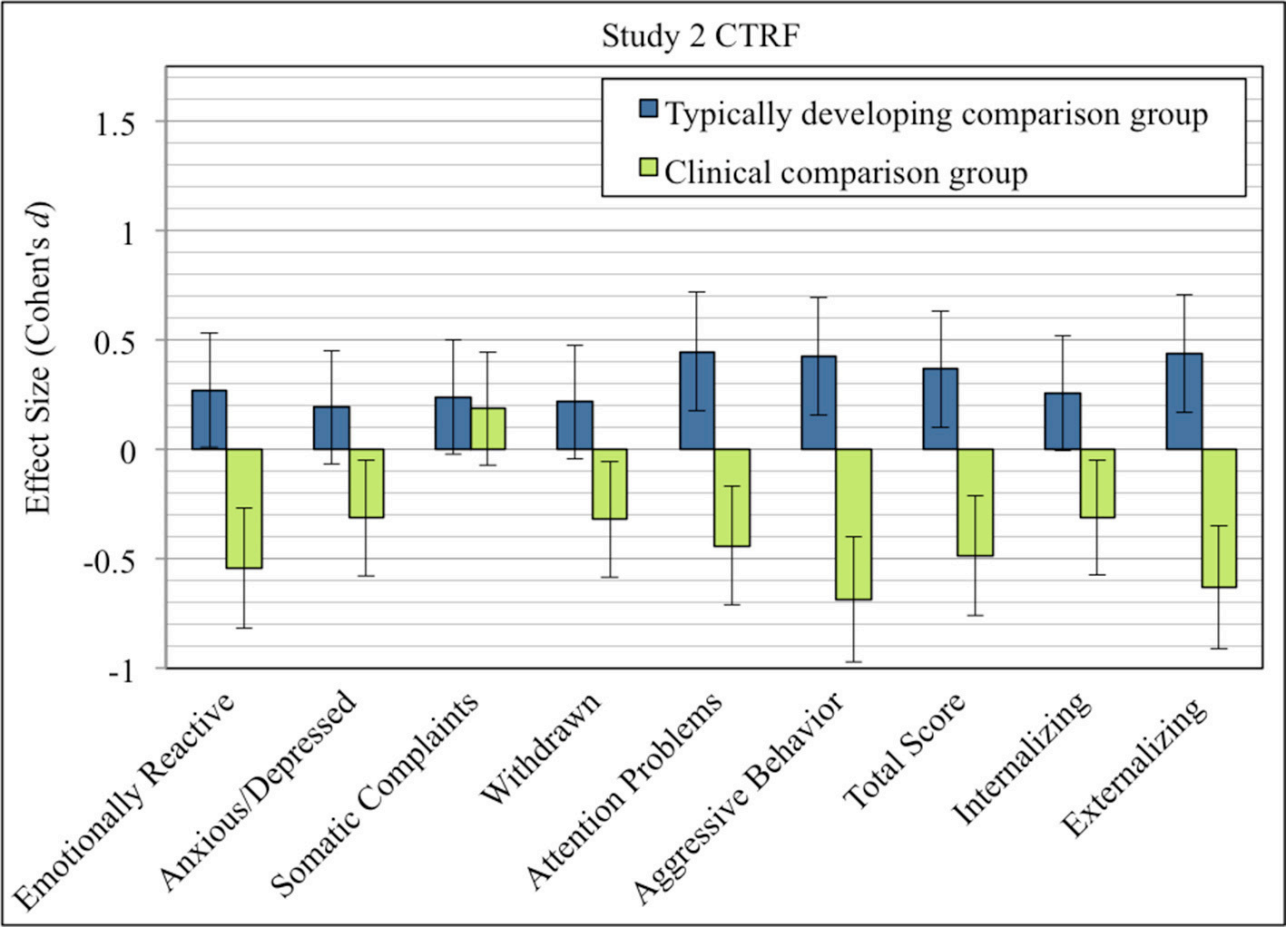
Effect sizes (Cohen's *d*) of CTRF syndrome scales comparing refugee children from Study 2 with typically developing children (blue), and clinically-referred children (green). Error bars indicate 95% confidence intervals of effect sizes.

Compared to clinical norm data, refugee children had lower scores on total problems, and lower scores on all scales with medium effect sizes except for the scale Somatic Complaints, where differences were only marginal (see Table 6). Prevalence rates indicate that about one fifth of the refugee children had externalizing problems in a clinical range and about one third in a subclinical range. However, as shown in Figure 3, only a small fraction of children showed clinically relevant behavior on specific syndrome scales. However, there was a tendency for syndrome manifestations to be higher when compared to typically developing children, but not when compared to clinically-referred children, except for Somatic Complaints.

**Table 6:** Study 2 CTRF syndromes of refugee children compared to clinical norm data of clinically-referred children.

**CTRF syndromes**	**Median (IQR) difference**	***z*-values**	***p*-values**	**Effect size *d* [CI 95%]**	**Clinical cutoff *n* (%)**	**Subclinical cutoff *n* (%)**
Emotionally Reactive	−2.4 (3.0)	−4.0	< 0.01	−0.5 [−0.3; −0.8]	4 (6.9)^a^	6 (10.3)^c^
Anxious/Depressed	−1.8 (4.0)	−2.9	0.04	−0.3 [−0.1, −0.6]	4 (6.9)^a^	6 (10.3)^c^
Somatic Complaints	−0.7 (1.3)	0.5	1.0	0.2 [0.1, −0.4]	5 (8.6)^a^	5 (8.6)^c^
Withdrawn	−3.4 (5.3)	−2.6	0.08	−0.3 [−0.1; −0.6]	4 (6.9)^a^	8 (13.8)^c^
Attention Problems	−2.7 (7.3)	−3.2	0.01	−0.4 [−0.2; −0.7]	4 (6.9)^a^	8 (13.8)^c^
Aggressive Behavior	−10.4 (13.3)	−4.3	< 0.01	−0.7 [−0.4, −1.0]	4 (6.9)^a^	10 (17.2)^c^
Total Score	−26.3 (44.7)	−3.8	< 0.01	−0.5 [−0.2; −0.8]	8 (13.8)^b^	17 (29.3)^d^
Internalizing	−6.7 (10.5)	−3.2	0.02	−0.3 [−0.1; −0.6]	8 (13.8)^b^	13 (22.4)^d^
Externalizing	−12.6 (20.5)	−4.1	< 0.01	−0.6 [−0.3; −0.9]	11(19)^b^	18 (31.0)^d^

CTRF, Caretaker Teacher Report Form; M, mean; SD, standard deviation; n, number of cases; r, point bivariate correlations coefficient; d, Cohen's d; Standardized z-values for one-sample Wilcoxon-test between refugee children and norm data of clinically-referred children (18), comparison of median difference to 0; P-values were adjusted for multiple comparisons using the Bonferroni correction; Confidence interval was calculated using the syntax of Wuensch (21);

a *Number of refugee children who scored above clinical cutoffs as proposed by Achenbach and Rescorla (18)*.

b *Number of refugee children who scored at least two standard deviations above the norm (18)*.

c *Subclinical cutoffs (18)*.

d *Number of refugee children who scored at least one standard deviation above the norm (18)*.

#### Discussion

Assessment of caretakers' views allowed us to incorporate a more comprehensive picture of children's mental health status. All syndrome scales were elevated into a positive direction when compared to data of typically developing children. Externalizing syndromes were especially elevated, with medium effect sizes. In contrast, only Somatic Complaints were increased in comparison to data of clinically-referred children. This provides further partial evidence of the potential mental health pattern proposed in the Pilot Study and Study 1. However, caretakers seemingly observe a wide range of psychological problems in refugee children. The fact that Attention Problems and Aggressive Behavior are the syndromes that are most elevated might reflect the significance of those behaviors for primary care settings.

## General discussion

The aim of the study was to explore the mental health status of recently-arrived, young refugee children. We screened for mental health problems in a pilot study and two main studies using applicable methods that have been adopted internationally and two sources of information, parents and caretakers. In all studies, syndrome manifestations were compared to norm data both of typically developing children and of clinically-referred children. In addition, prevalence rates were reported, and comorbidities were calculated for the most pronounced syndrome scales, which revealed first evidence of a potential refugee-specific mental health pattern.

Comparing the reported clinical manifestations of Study 1 and Study 2, two results become the subject of discussion. First, parents reported, on average, more mental health problems than caretakers. Still, the results are not contradictory, as effect sizes across all syndrome scales indicate similar results. Two syndrome scales (Attention Problems and Aggressive Behavior) showed a similar effect size in both studies. The high concordance between the groups of informants on these scales underscores the clinical relevance of our results.

Second, parents reported more internalizing problems, whereas caretakers stressed externalizing problems. It is possible that a systematic bias influenced the concordance between the ratings of parents and caretakers. According to Achenbach and Rescorla (18), parents report internalizing problems with a higher frequency than caretakers, whereas the reverse is true for externalizing problems. In our sample, parents spent significantly more time with their children compared to caretakers, who take care of a group of children for only a few hours a day [for a description of early childhood care centers see Busch et al. (under review)]. Additionally, language barriers may hamper communication between children and caretakers. Hence, children may communicate internalizing problems in a language that caretakers do not understand. The scores on the CTRF may, therefore, underestimate the true manifestations of emotional and behavioral difficulties.

As parents' psychological burden is likely high as well, the accuracy of the ratings for syndrome scales such as “Anxious/Depressed” may be distorted as a consequence (23, 24). A difference between the syndromes could also point to real differences in psychological functioning across situations. In fact, some disorders, such as ADHD, are known to be highly susceptible to situational contexts (25). These rater biases might explain why data from Study 2 was not able to fully confirm the potential refugee-specific pattern found in the Pilot Study and Study 1.

A growing body of research on refugee children worldwide reported elevated prevalence rates of mental health concerns (6–8). A meta-analysis, which included 17 studies on refugee children, found that refugee children have moderately poorer mental health outcomes compared to non-refugee children (26). However, research on young refugee children in Germany is still scarce, which comes as a surprise, as a great number of families with young children has claimed asylum within the last few years (3). Among the first, Soykoek et al. (11) investigated prevalence rates of PTSD among refugee children from 0 to 6 years of age in Germany. Unlike Soykoek et al. (11), we did not conduct clinical interviews for PTSD, but used screening instruments, which allowed us to check for a broad range of clinical syndromes. Each study takes a different perspective when describing similar phenomena, and thus they can be viewed as complementary. While both studies found elevated rates of PTSD/signs of PTSD (26% vs. 37% in our study), we also found various internalizing and externalizing behavioral problems. This suggests that researchers and clinicians should broaden their focus and be aware that refugee children exhibit a variety of mental health problems apart from PTSD.

Considering the fact that recently arrived refugee children are exposed to a large number of specific risk factors (5), we expected to find a specific early childhood mental health pattern in refugee children. The mental health status on the scales Anxious/Depressed, Withdrawn, and Attention Problems were in a clinical range, whereas mental health status on the remaining scales was better than in a clinical comparison group. Although the syndromes mostly occurred individually, about one quarter of children exhibited them in a comorbid fashion. Thus, parent reports delivered a first hint for a refugee-specific mental health pattern.

Research on specificity regarding the development of psychopathologies yields mixed results (12). Even less is known about the effects of war-related stressors on the development of specific mental health problems. One study on school children reported differences regarding the mental health status, depending on the experience of violence in the context of war (17). Refugee children might show a predominance of internalizing symptoms that differs from other traumatized groups (27). The authors hypothesized that this pattern might be a consequence of acute stress in the context of armed conflicts. However, to confirm the notion of specificity, two conditions have to be met: Namely, a specific risk factor (condition 1) must lead to a specific mental health outcome (condition 2) (12).

Two main approaches can be distinguished that describe the mechanisms of how risk factors lead to negative mental health outcomes. According to the cumulative risk approach, each risk factor has an independent additive effect on children's mental health outcomes (28). It is often assumed that negative experiences accumulate until they reach a certain threshold (29). Once the threshold is exceeded, children's mental health outcomes deteriorate rapidly.

A dimensional approach proposed by McLaughlin and Sheridan (30) differentiates between risk factors along two dimensions, threat and deprivation. Threat is defined as an event involving harm (e.g., community violence), whereas deprivation is defined as experiences involving an absence of expected inputs (e.g., neglect). The development of a specific kind of psychopathology may depend on the dimension of risk factors involved. There is evidence that refugee children and their families are not only subject to threatening events, but also to severe deprivation, such as a lack of food, isolation, and a lack of shelter (31). Thus, the refugee-specific mental health pattern might well be interpreted in the context of a dimensional risk approach.

The second condition of specificity relates to mental health outcomes. Research has repeatedly shown that young children may develop PTSD, but symptoms differ in some aspects from those of older populations (32, 33). One third of the refugee children in Study 1 showed signs of PTSD. Although risk factors might differ in severity and type from other traumatized groups, they might still facilitate the development of PTSD. Furthermore, Hodges et al. (15) showed that the exposure to multiple, different traumatic events leads to a higher complexity of symptoms. This complements our result of a wide range of affected syndromes. However, it is also possible that some refugee children suffer from PTSD, whereas others develop a mental health pattern that is specific to the refugee population. Our explorative study cannot provide an answer to these questions, but can rather be viewed as a starting point for novel specificity research on refugee children.

It is of particular importance to study the effects of traumatization in early childhood, as early experiences may have different negative effects on future outcomes compared to later traumatization. According to Maercker et al. (34), experiencing traumatization in childhood, before the age of 12, increases the risk of suffering from major depression, whereas traumatization beyond the age of 12 increases the risk for PTSD. The authors speculated that the development of PTSD might require a high degree of maturation of the brain in order to be able to organize intrusive memories and process fear stimuli. This may not be possible in young children. However, early exposure to traumatic events may have sensitizing effects on children by causing a dysfunction of the physiological stress axis. This is likely to make children more prone to various psychopathological signs and symptoms over the course of their development (29). Apart from developmental factors, young refugee children may be particularly vulnerable to behavioral difficulties because caregivers were exposed to high levels of stress as well. The onset of mental health concerns may even be rooted in stressors regarding the prenatal environment. About one quarter of mothers in our first study reported that they were pregnant with the child during their flights. Stressors during pregnancy are likely to have long-lasting effects on children (35, 36). This underscores that the effects of traumatization in an early stage of the development and the high-risk environment of refugees are difficult to disentangle.

Due to their explorative nature and practical obstacles, our studies come with some limitations. The feasibility of the instrument was checked in a pilot study. Nevertheless, a selection bias could not be fully avoided. The mean duration of schooling in our sample in Study 1 was nearly 10 years. This is about twice as long as the average time of schooling in Syria before the war (United Nations Human Development Reports). This might hamper the generalizability of our sample. The possibility of a cultural bias, particularly for the parents' report, cannot be fully excluded either. The CBCL is found to be cross-culturally valid, and norm values between children from the United Arab Emirates and the U.S. have not shown substantial differences (20). Still, cross-cultural validation, particularly with people from Syria and Iraq, is needed. To assess signs of post-traumatic stress, we relied on a PTSD scale derived from CBCL items. This scale shows sufficient sensitivity (19), and is more reliable than comparable PTSD scales derived from the CBCL (37). However, all these PTSD scores derived from the CBCL should be interpreted with care, as they may lack the specificity to differentiate between PTSD and other psychiatric disorders like communication disorders, disruptive behavior disorders, or other anxiety disorders (38, 39).

Refugees share a number of risk factors, and are thus described as a vulnerable population in need of specific research and intervention programs (5). Nevertheless, the similar risk factors should not mask the heterogeneity within the refugee group. A meta-analysis revealed that mental health problems differed substantially between studies because of contextual factors before and after displacement (26). For example, the authors reported that effect sizes depended on economic opportunities and differed between those who were displaced internally and those who were displaced externally. Hence, in order to understand underlying causes of mental health problems in more detail, contextual factors should be addressed in future studies. For young children in particular, parental mental health status potentially constitutes a crucial context for the development of mental health problems (40).

Our studies deliver evidence for mental health problems of young refugee children in Germany. While a significant percentage of children showed signs of post-traumatic stress, we found a broad scope of mental health domains that were affected. Researchers should therefore not exclusively narrow their focus on PTSD symptomatology, but encompass other mental health problems as well. The question of whether or not the striking manifestations of anxiety, depression, withdrawal behavior, and attention problems resemble a refugee-specific syndrome pattern in young children cannot be answered at this point in time. Future studies should follow these hints and explore the mental health pattern in more detail. Revealing a specific pattern is an important step in early diagnosis of mental health concerns in this vulnerable group, in offering tailored intervention, and in preventing the onset of mental disorders as early as possible.

## Ethics statement

This study was carried out in accordance with the recommendations for psychological research of the Deutsche Gesellschaft für Psychologie (DGP) with written informed consent from all subjects. All subjects gave written informed consent in accordance with the Declaration of Helsinki. The protocol was approved by the ethical committee of the Faculty of Psychology at the Ruhr-University Bochum.

## Author contributions

TB contributed to the conception and design of the study, to the analysis and interpretation of data, and to drafting the manuscript. HL was involved in drafting the manuscript and contributed to the interpretation of the data. JB was involved in the conception of the study and revised the manuscript. RK revised the manuscript and contributed to the interpretation of the data. BL made contributions to the conception of the study, revised the manuscript, and gave final approval of the version to be published.

### Conflict of interest statement

The authors declare that the research was conducted in the absence of any commercial or financial relationships that could be construed as a potential conflict of interest.
